# The Preclinical Pharmacology of Tepotinib—A Highly Selective MET Inhibitor with Activity in Tumors Harboring *MET* Alterations

**DOI:** 10.1158/1535-7163.MCT-22-0537

**Published:** 2023-03-30

**Authors:** Joachim Albers, Manja Friese-Hamim, Anderson Clark, Oliver Schadt, Gina Walter-Bausch, Christopher Stroh, Andreas Johne, Niki Karachaliou, Andree Blaukat

**Affiliations:** 1 Research Unit Oncology, the healthcare business of Merck KGaA, Darmstadt, Germany.; 2 Corporate Animal Using Vendor and Vivarium Governance (SQ-AV), Corporate Sustainability, Quality, Trade Compliance (SQ), Animal Affairs (SQ-A), the healthcare business of Merck KGaA, Darmstadt, Germany.; 3 Research Unit Oncology, EMD Serono Research and Development Institute, Inc., Billerica, Massachusetts.; 4 Global Medicinal Chemistry, the healthcare business of Merck KGaA, Darmstadt, Germany.; 5 Clinical Biomarkers and Companion Diagnostics, the healthcare business of Merck KGaA, Darmstadt, Germany.; 6 Global Clinical Development Unit, the healthcare business of Merck KGaA, Darmstadt, Germany.

## Abstract

The mesenchymal–epithelial transition factor (*MET*) proto-oncogene encodes the MET receptor tyrosine kinase. *MET* aberrations drive tumorigenesis in several cancer types through a variety of molecular mechanisms, including *MET* mutations, gene amplification, rearrangement, and overexpression. Therefore, MET is a therapeutic target and the selective type Ib MET inhibitor, tepotinib, was designed to potently inhibit MET kinase activity. *In vitro*, tepotinib inhibits MET in a concentration-dependent manner irrespective of the mode of MET activation, and *in vivo*, tepotinib exhibits marked, dose-dependent antitumor activity in MET-dependent tumor models of various cancer indications. Tepotinib penetrates the blood–brain barrier and demonstrates strong antitumor activity in subcutaneous and orthotopic brain metastasis models, in-line with clinical activity observed in patients. *MET* amplification is an established mechanism of resistance to EGFR tyrosine kinase inhibitors (TKI), and preclinical studies show that tepotinib in combination with EGFR TKIs can overcome this resistance. Tepotinib is currently approved for the treatment of adult patients with advanced or metastatic non–small cell lung cancer harboring *MET* exon 14 skipping alterations. This review focuses on the pharmacology of tepotinib in preclinical cancer models harboring *MET* alterations and demonstrates that strong adherence to the principles of the Pharmacological Audit Trail may result in a successful discovery and development of a precision medicine.

## Introduction

**Figure 1.**
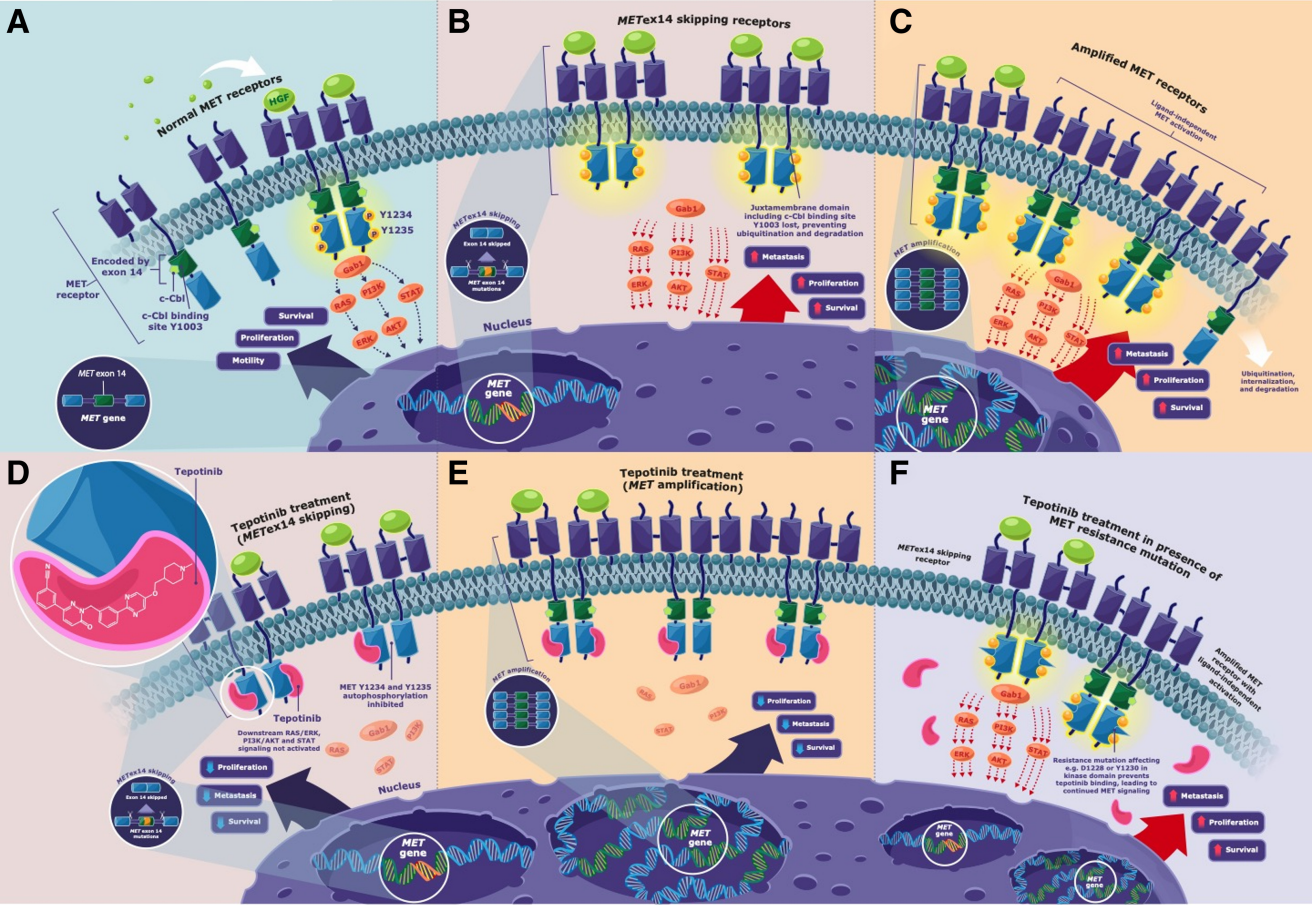
Simplified diagrammatic schema showing **A,** In a physiologically normal context, MET signaling is activated when the HGF ligand binds to the extracellular domain of the MET receptor that induces homodimerization and stimulates auto-phosphorylation of the tyrosine residues Y1234 and Y1235 in the cytoplasmic regions of the receptor. This leads to activation and recruitment of the adaptor/scaffold protein Gab1 and activation of downstream signaling pathways (including RAS/ERK, PI3K/AKT, and STAT), resulting in cell survival, proliferation, and motility. Under normal physiological conditions there is a balance between MET signaling and downregulation of MET (3, 4). In addition to MET signaling from the interaction between the ligand and the MET receptor, there have been published reports of MET internalization promoting additional signaling whereby the formation of early MET-containing endosomes can (i) trigger the activation of ERK leading to focal adhesions of the phosphorylated ERK that may mediate HGF-induced cell migration, or (ii) MET may be trafficked along the microtubule network and accumulate in a perinuclear compartment where it may trigger the phosphorylation of STAT3 leading to the translocation of the phosphorylated STAT3 into the nucleus, which could induce downstream signaling (3–5). Publications have also reported the downregulation of MET may occur through (i) endocytosis of MET and the formation of multivesicular bodies leading to MET undergoing lysosomal degradation, or (ii) MET degradation may occur through sequential proteolytic cleavage at the juxtamembrane site, with the cleaved intracellular MET fragment being destroyed in the proteasome, whereas the cleaved extracellular MET may generate an extracellular “decoy MET” that could capture the HGF ligand and interfere with other intact MET receptors (3, 4). **B,** Dysregulation of the MET pathway can occur through several mechanisms including alterations in the *MET* gene, such as *MET*ex14 skipping (*MET*ex14 encodes the MET receptor juxtamembrane domain, which contains negative regulatory elements such as the Y1003-binding site for c-Cbl E3 ubiquitin ligase, which under normal conditions would facilitate the ubiquitination of the MET receptor, resulting in the internalization, trafficking to late endosomes and degradation. However, in tumors harboring *MET*ex14 skipping, the receptor is truncated and the loss of this binding site for c-Cbl E3 ubiquitin ligase results in reduced ubiquitination and degradation of the receptor, through decreased lysosomal receptor degradation, leading to sustained MET signaling that can promote uncontrolled proliferation, survival, and metastasis. **C,** Dysregulation of the MET pathway can also occur through *MET*amp (where the increase in the *MET* copy number results in increased synthesis of MET, leading to increased MET signaling and subsequent increased cell proliferation, survival, and metastasis). Frazier et al. have reported ligand-independent phosphorylation of receptor tyrosine kinases (RTK) in cancer cells with *MET*amp, where co-localization of MET and RTKs can occur in the Golgi apparatus, and the researchers postulated that when MET is overexpressed, it may accumulate in the Golgi apparatus and this overcrowding facilitates the nonspecific interaction between MET and newly synthesized RTKs (during the RTK trafficking to the plasma membrane) leading to the premature phosphorylation of RTKs and their subsequent downstream effect (5). In tumors harboring *MET*ex14 skipping (**D**) or *MET*amp (**E**), the MET inhibitor tepotinib binds to the kinase domain and blocks the autophosphorylation of the intracellular domain of the MET receptor, thereby impeding the activation of the downstream signaling pathways (including RAS/ERK, PI3K/AKT, and STAT), and inhibiting tumor cell proliferation, survival, and metastasis. **F,** Secondary MET kinase domain mutations affecting for example Y1230 and D1228 prevent the binding of tepotinib to the MET receptor, leading to continued MET signaling. AKT, protein kinase B; c-Cbl, Casitas B-lineage lymphoma; ERK, extracellular signal-regulated kinase; Gab1, Grb2-associated binder 1; HGF, hepatocyte growth factor; MET, mesenchymal–epithelial transition proto-oncogene; *MET*amp, *MET* amplification; *MET*ex14, *MET* exon 14; PI3K, phosphoinositide 3-kinase; RAS, rat sarcoma viral oncogene; STAT, signal transducer and activator of transcription.

The mesenchymal–epithelial transition factor (*MET)* proto-oncogene, which is located on chromosome 7q21–31, encodes the MET receptor tyrosine kinase (1). Activation of MET, through binding of hepatocyte growth factor (HGF) to the extracellular domain, stimulates auto-phosphorylation of several tyrosine residues, triggering downstream activation of the rat sarcoma viral oncogene (RAS)/MAPK, PI3K–protein kinase B (Akt), and STAT signaling pathways (Fig. 1A; refs. 1–5). In cancer, MET pathway activation promotes cell proliferation, survival, angiogenesis, migration, and invasion (1, 6). MET is a therapeutic target in several cancers, including non–small cell lung cancer (NSCLC), hepatocellular carcinoma (HCC), and gastric cancer (1, 7). MET activation is associated with a poor prognosis and resistance to standard-of-care anticancer treatments (1, 7).

MET pathway dysregulations occur through alterations in the *MET* gene, such as *MET* exon 14 (*MET*ex14) skipping (Fig. 1B) or *MET* amplification (*MET*amp; Fig. 1C; refs. 1, 2). *MET*ex14 encodes the juxtamembrane domain of MET, which contains various negative regulatory sites, including: the protein kinase C (PKC) phosphorylation site Ser985; Tyr1003, which is phosphorylated to activate recruitment of the E3 ubiquitin ligase Casitas B-lineage lymphoma (CBL); and a caspase cleavage site involved in apoptosis (1, 8). Because Ser985 and PKC control cellular HGF responsiveness, *MET*ex14 skipping can desensitize to PKC-induced inhibitory signals, thereby increasing MET activation (8). Similarly, *MET*ex14 skipping results in MET receptors lacking the ubiquitin-binding site Tyr1003, leading to escape of the receptor from lysosomal degradation and its recycling to the surface and, hence, sustained MET activation (1, 9). Because caspase cleavage leads to MET inactivation and generates a pro-apoptotic receptor fragment, *MET*ex14 skipping also results in loss of this negative regulatory mechanism (8). Consequently, *MET*ex14 skipping alterations cause oncogenic MET activation by expression of a truncated receptor with increased stability, and augmented and prolonged signaling capability (1, 8). *MET*ex14 skipping occurs in approximately 3%–4% of lung adenocarcinomas, in approximately 2% of squamous cell lung cancers, with a higher incidence of approximately 8%–30% in pulmonary sarcomatoid carcinoma (1, 2, 10), and may be more common in lung cancer brain metastases than primary lung tumors (11). Besides NSCLC, *MET*ex14 skipping has only rarely been noted in other solid tumors, including brain glioma (12), although a higher prevalence has been reported in secondary glioblastoma (13).

*MET*amp is thought to be a mechanism of overexpression of the MET receptor and its constitutive, ligand-independent activation, thereby dysregulating the MET pathway and promoting tumor growth (1, 2). *De novo* high-level *MET*amp occurs in approximately 1%–2% of NSCLCs and has been identified as a primary oncogenic driver (1, 14–16). *MET*amp is also detectable in 8%–14% of tumors with *MET*ex14 skipping (17, 18). *MET*amp manifests in up to 30% of patients with acquired resistance to epidermal growth factor receptor (EGFR) tyrosine kinase inhibitors (TKI; refs. 19) and is reported in approximately 15% of patients with resistance to ALK, RET or ROS1 inhibitors (20, 21).

Other mechanisms of MET dysregulation include *MET* fusions and activating *MET* kinase domain mutations (22). *MET* fusions are rare, may be a primary oncogenic driver in NSCLC, and are also reported in other cancers, including gastric cancer and glioma (23). Activating *MET* kinase domain mutations primarily occur in papillary renal-cell carcinoma, including the hereditary type 1 form (24).

Molecularly targeted cancer therapeutics have transformed cancer management, with departure from a “one-size-fits-all” approach and increasing focus on precision medicine. Although the oncogenic role of MET was established >30 years ago, early attempts to develop MET inhibitors were hampered by inadequate pharmacologic potency and/or selectivity, and suboptimal target population selection for clinical testing [for further information, please see Schadt and colleagues (ref. 25) and Wu and colleagues (ref. 2) and references therein]. Recently, several selective MET-targeted small-molecule TKIs have been developed, of which three – tepotinib hydrochloride hydrate (hereafter “tepotinib”), capmatinib, and savolitinib – have been approved on the basis of clinical benefit for patients with advanced/metastatic NSCLC harboring *MET*ex14 skipping alterations (26, 27).

Considering the principles of the Pharmacological Audit Trail (PhAT; ref. 28), this review focuses on key preclinical pharmacology data generated during the discovery and development of tepotinib, as an orally available and highly selective MET TKI, and precision medicine targeting oncogenic *MET* alterations (10, 29). Furthermore, we summarize ongoing clinical development and discuss potential mechanisms of pre-existing and acquired tepotinib resistance.

## *In Vitro* Pharmacology of Tepotinib

Tepotinib is a highly selective, type Ib, ATP-competitive, small-molecule MET inhibitor (Fig. 1D and E). It binds to MET in a U-shaped conformation by interacting with Y1230, D1222, and M1160 in the hinge region (2, 22, 30, 31).

### Tepotinib is a potent and highly selective MET inhibitor in biochemical assays

Tepotinib inhibition of MET kinase activity was analyzed in biochemical flash-plate assays using a His6-tagged recombinant human MET kinase domain (amino acid residues 974-end), [γ^33^P]-labeled ATP and a biotinylated peptide substrate (biotin-poly-AlaGluLysTyr, 6:2:5:1; ref. 30). Tepotinib inhibited MET kinase activity in a concentration-dependent manner with IC_50_ values of 1.7 and 1.8 nmol/L, respectively, in two independent experiments (30, 32, 33).

Selective type Ib MET inhibitors reduce the risk of off-target effects and are likely to have a superior safety profile compared with multi-kinase inhibitors, which is critical for treating biomarker-selected patients with monotherapy and especially with combination treatments (2, 10). Tepotinib was therefore designed and optimized to selectively inhibit MET and avoid poly-pharmacology. Overall, tepotinib selectivity was tested against >400 kinases and kinase variants, other than MET and mutant MET versions, at clinically relevant concentrations and above (32, 33). A tepotinib concentration of 0.1 μmol/L corresponds to approximately 200% of the free steady-state maximum concentration of tepotinib of 52 nmol/L in patients with the 500 mg once daily (QD) clinical dose. At this clinically relevant concentration, MET was completely inhibited (≥99%) whereas very few of the >300 MET-unrelated kinases were weakly inhibited, with tropomyosin receptor-kinase A (TrkA) being the most strongly inhibited (35%) non-MET kinase (33). Tepotinib selectivity was also measured at supratherapeutic concentrations of 1 and 10 μmol/L, corresponding to approximately 19- and 190-fold, respectively, of the average free steady-state maximum tepotinib concentration with 500 mg QD (33). Although tepotinib completely inhibited MET (100%) at 1 μmol/L, the strongest inhibition of a MET-unrelated kinase at this concentration was observed for TrkC (91%) and TrkA (70%) in panels of 399 and 305 kinases, respectively. At 10 μmol/L, the strongest inhibition was observed for TrkB (94% in a panel of 36 kinases from which TrkB was the only Trk family member; ref. 33). The high selectivity of tepotinib was further confirmed by an independent study analyzing 243 kinase inhibitors using a chemical kinome screen (Kinobeads) in tumor cell lysates, in which tepotinib did not interact with any non-MET kinase at up to 1 μmol/L (33, 34).

As TrkA was the MET-unrelated kinase most strongly inhibited by tepotinib at 0.1 μmol/L, and TrkA (NTRK1) is a relevant target in NSCLC, an *in vitro* study evaluated whether tepotinib at ≥0.1 μmol/L had antitumor activity against TrkA (NTRK1)-dependent, TPM3-NTRK1–expressing, colorectal cancer KM-12 cells (33). Test inhibitors included tepotinib, crizotinib [a multi-kinase inhibitor of MET (type Ia), ALK, and ROS1], and the NTRK inhibitors larotrectinib and entrectinib (entrectinib is a pan-Trk inhibitor of TrkA, TrkB and TrkC, with activity against ROS1 and ALK; refs. 1, 35, 36). In this study, entrectinib, larotrectinib, or crizotinib markedly decreased cellular viability with IC_50_ values ranging from 1 to 8 nmol/L, 9 to 22 nmol/L, and 55 to 112 nmol/L (33), respectively, in line with published IC_50_ values for entrectinib and larotrectinib in the low nanomolar range (35). In contrast, up to 1.3 μmol/L, tepotinib did not have antitumor activity in the same TrkA (NTRK1)-dependent KM-12 cells and, even at the highest supratherapeutic concentration (5 μmol/L), only a 50% reduction of cellular viability was observed (33). These findings corroborate the high selectivity of tepotinib and support the concept that partial, incomplete inhibition of unrelated kinases is insufficient for robust antitumor activity, particularly at clinically relevant concentrations (33).

### Tepotinib is a potent inhibitor of MET in tumor cells, irrespective of the mode of MET activation

On the basis of the PhAT, one of six important aspects for successful drug development is to define the potential patient population early (28). Consistent with this concept and based on the high selectivity of tepotinib, the drug-discovery phase of tepotinib development placed a strong emphasis on biomarker-driven selection of tumor models for *in vitro* and *in vivo* pharmacology studies. Another critical aspect of the PhAT is the identification of a pharmacodynamic (PD) biomarker with close functional proximity to the target, and a robust assay to measure modulation of this biomarker. As has become standard in MET inhibitor development, MET autophosphorylation on Y1234 and Y1235 was established as a suitable proximal PD biomarker, and was used to successfully define the extent of target modulation needed to achieve antitumor efficacy with tepotinib preclinically and later clinically (30, 37, 38). Use of tumor pY^1234/1235^MET/total MET ratio as a PD biomarker to guide tepotinib dose selection was further supported in an independent study using a *MET*-amplified gastric cancer xenograft model (SNU-5; ref. 39).

**Figure 2.**
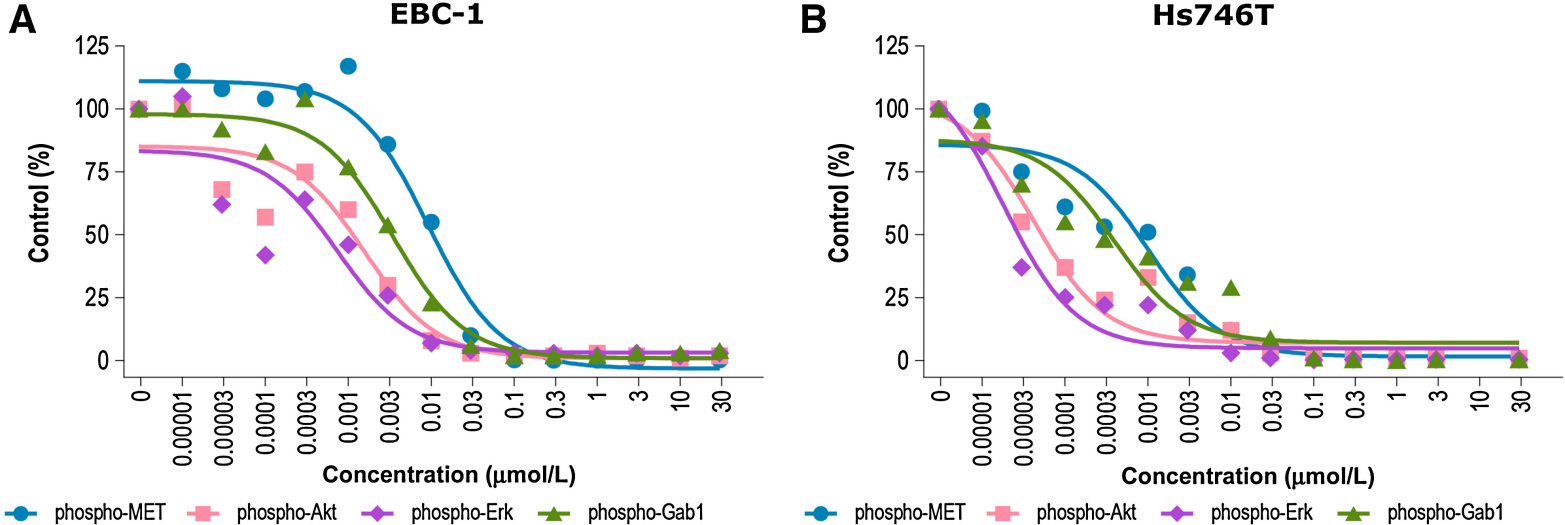
Inhibition of MET phosphorylation and downstream signaling molecules with tepotinib in EBC-1 lung cancer cells (**A**; ref. 30) and Hs746T gastric cells (**B**; ref. 33). **A,** Adapted with permission from Bladt F, et al. Clin Cancer Res 2013;19 (11):2941–51. AKT, protein kinase B; ERK, extracellular signal-regulated kinase; Gab1, Grb2-associated binder 1; MET, mesenchymal–epithelial transition factor.

*In vitro*, in EBC-1 lung cancer cells harboring high-level *MET*amp, tepotinib potently inhibited MET phosphorylation (IC_50_ = 1.1 nmol/L), as measured using a capture Enzyme-linked Immunosorbent Assay (30, 33). Strong MET inhibition was also observed in the gastric cancer cell lines Hs746T (harboring *MET*ex14 skipping and high-level *MET*amp) and GTL-16 (harboring high-level *MET*amp), with IC_50_ values of 2.5 and 2.9 nmol/L, respectively (33). To assess the effect of tepotinib-mediated MET inhibition on downstream signal transduction, phosphorylation of MET Y1234/1235, Grb2-associated binder 1 (Gab1) Y627, Akt S473, and ERK1/2 T202/Y204 was analyzed in EBC-1 cells using phospho-site–specific antibodies (Fig. 2A; refs. 30, 33). Inhibition of MET phosphorylation by tepotinib in the low nanomolar range was confirmed (IC_50_ = 9.2 nmol/L), and phosphorylation of the downstream adaptor/scaffold protein Gab1 was inhibited with an IC_50_ value of 3.4 nmol/L. Activation of anti-apoptotic Akt signaling and ERK phosphorylation was also efficiently inhibited in EBC-1 cells with low or sub-nanomolar IC_50_ values (30, 33). Similar results were observed in Hs746T cells (Fig. 2B; ref. 33). A549 cells were used to study tepotinib effects on HGF-dependent MET phosphorylation (33), because these cells do not harbor an oncogenic *MET* alteration and require HGF stimulation to activate MET (40). To measure the effect of tepotinib on HGF-mediated MET phosphorylation, cells were incubated in serum-free medium for 20 hours, pretreated with tepotinib in serum-free medium for 45 minutes, and stimulated with 100 ng/mL HGF for 5 minutes (33). As in the HGF-independent models, tepotinib showed potent and concentration-dependent MET kinase inhibition, with an IC_50_ value of 5.4 nmol/L (30, 33).

Tepotinib has a fast cellular uptake and long retention in tumor cells, as demonstrated by washout experiments (30). A549 cells were treated for 45 minutes with tepotinib and then incubated in tepotinib-free medium for 14 hours, before stimulation with 100 ng/mL HGF (30). The 45-minute incubation with tepotinib was sufficient to completely inhibit HGF-induced MET phosphorylation 14 hours after withdrawal of tepotinib, with an IC_50_ value of 5.3 nmol/L (30, 33), demonstrating rapid uptake and cellular retention of tepotinib, with long-lasting MET inhibition. Persistence of tepotinib inhibition under washout conditions may be explained by the long residence time of tepotinib and its retention in the lysosomal compartment to provide a local drug reservoir (41). Furthermore, 10% (v/v) serum only moderately affected tepotinib inhibition of HGF-induced MET phosphorylation in A549 cells (average IC_50_ values of 21 and 23 nmol/L with murine and human serum, respectively; ref. 30), suggesting that tepotinib activity will be largely maintained under whole-blood conditions *in vivo*.

### Tepotinib selectively inhibits proliferation of MET-dependent tumor cells in 2D and 3D cell culture

MET promotes tumor cell proliferation and survival, particularly in cells with *MET*amp (30). To investigate the effect of tepotinib on tumor cell viability *in vitro*, MKN-45 (*MET*amp) and SNU-16 gastric cancer cells (non-*MET*amp) were incubated with increasing concentrations of tepotinib, and cellular metabolic activity was assessed (30). Whereas incubation with tepotinib for 48 hours considerably inhibited MKN-45 cell viability (IC_50_ = 6 nmol/L), SNU-16 cells were less sensitive (IC_50_ = 3 μmol/L), thus demonstrating a selective inhibitory effect of tepotinib on *MET*amp cells (30, 33). In another study, tepotinib had the highest MET inhibitory activity among 1,449 FDA-approved agents in *MET*amp SNU-620 gastric cancer cells (42). Further studies showed concentration-dependent inhibition of *MET*amp SNU-620 and MKN-45 cell growth by tepotinib, with average IC_50_ values of 9 and 7 nmol/L, respectively (42). *In vitro* wound-healing tests evaluating the effect of tepotinib, with or without HGF, on closure of a gap induced by scratching a layer of NCI-H441 lung cancer cells demonstrated that tepotinib inhibits cancer cell migration at clinically relevant concentrations (30).

Anchorage-independent growth is a hallmark of anoikis resistance and the path to invasive, metastatic tumor growth. To mimic the ability of cancer cells to grow in an anchorage-independent three-dimensional manner, murine NIH-3T3 cells co-transfected with human HGF and MET were cultured in a three-dimensional soft agar matrix (33). Treatment of established colonies with tepotinib for 5 days caused dose-dependent growth inhibition (IC_50_ = 1.8 nmol/L; ref. 33).

## *In Vivo* Pharmacology of Tepotinib

### PD activity of tepotinib

According to the PhAT, establishing the pharmacokinetic (PK) profile of a drug and understanding the PK/PD relationship in preclinical *in vivo* models is essential (28, 30, 32, 43). The tepotinib PK/PD relationship was first investigated in a single-administration study, using the Hs746T gastric cancer model (harboring *MET*ex14 skipping and *MET*amp) with constitutive MET phosphorylation (30, 33). Consistent with the high volume of distribution of tepotinib of >8 L/kg in mice, tepotinib concentrations were greater in the tumor than plasma at all tested doses (30). At oral doses ≥10 mg/kg, tumor drug concentrations were within the active pharmacological range identified *in vitro* (i.e., one-digit nanomolar IC_50_ values) at all time points tested (3–96 hours; ref. 30). The lowest tested tepotinib dose (3 mg/kg) induced transient MET phosphorylation inhibition, which peaked (90%) at 6 hours and decreased (to 30%) at 24 hours (30). In contrast, the other doses tested (10, 30, and 100 mg/kg) resulted in >90% inhibition of MET phosphorylation for ≥72 hours, in line with the long cellular retention of tepotinib *in vitro* (30).

### Tepotinib has strong antitumor activity in MET-dependent tumor models

*In vivo*, the antitumor efficacy of tepotinib was tested in mice bearing human tumor xenografts, representing various mechanisms of MET activation across multiple indications, including NSCLC and gastric cancer (30, 33, 37, 44). The dose-dependent inhibition of MET phosphorylation by tepotinib generally translated into dose-dependent antitumor activity in MET-dependent subcutaneous xenograft models, thus demonstrating preclinical proof of concept (30, 37). In mice bearing HGF-independent subcutaneous EBC-1 tumors with *MET*amp, tepotinib (free base) 15 mg/kg QD led to tumor growth inhibition, with complete regressions in 7/10 animals, whereas 25 mg/kg QD led to complete regression in 10/10 mice (30, 33). In mice bearing HGF-independent Hs746T xenografts with *MET*ex14 skipping/*MET*amp, effective tumor growth inhibition and regression, respectively, were observed at 3 and 6 mg/kg QD (30, 33).

Dose-dependent tepotinib antitumor activity was also observed *in vivo* in tumors with *MET* fusions (33). In one study, tepotinib induced strong tumor growth inhibition in subcutaneously implanted *TPR-MET*–transformed NIH-3T3 tumor cells at 12.5 mg/kg QD, and induced complete tumor regression in 4 out of 8 cases at 25 mg/kg QD (33, 43). Anecdotal evidence that patients with cancers with *MET* fusions may benefit from MET inhibitors comes from a case report of a woman with NSCLC and brain metastases harboring an *HLA–DRB1–MET* fusion. Following recurrence/progression on previous treatments, the patient received tepotinib and showed complete response in the brain, lung, and liver, which was sustained for almost 9 months (45).

In addition, tepotinib has demonstrated antitumor activity in preclinical models of liposarcoma (46), HCC (37, 47), head and neck squamous cell carcinoma (also showing a radiosensitization effect of tepotinib; ref. 48), bladder cancer (49), and neuroblastoma (50).

## Tepotinib Is a Brain-Penetrating Molecule with Intracranial Antitumor Activity

The ability of tepotinib to cross the blood–brain barrier (BBB) was assessed in Wistar rats (44). After intravenous infusion of tepotinib for 24 hours, the average brain-to-plasma ratio was 2.87 at steady state (44). Because of its considerably higher brain tissue protein-binding versus plasma protein-binding, the partition coefficient of unbound drug in plasma and brain (*K*_p u,u_) was 0.25, which would allow for intracranial target inhibition (44). This suggests that tepotinib is not freely diffusible but exhibits brain penetration (44). Brain penetration of tepotinib compares favorably with available data for capmatinib (brain-to-plasma ratio of 0.09 in rats) and crizotinib (cerebrospinal fluid-to-plasma ratio of 0.0006–0.003 in patients with brain metastases; refs. 51, 52).

In 20 subcutaneously implanted lung cancer brain metastasis patient-derived xenograft models, tepotinib at a suboptimal oral dose of 30 mg/kg QD (mimicking BBB-restricted drug exposure in the brain compartment) caused regression in 2/20 models (LU5349, −12%; LU5406, −88%; ref. 44). Molecular profiling revealed that only these two responding models had high-level *MET*amp, confirming the selective efficacy of tepotinib in tumors with oncogenic *MET* alterations (44). The identified tepotinib-sensitive *MET*amp NSCLC brain metastasis models were used to further evaluate the antitumor efficacy of tepotinib in a subcutaneous and orthotopic (intracranial) setting at a clinically relevant dose (125 mg/kg QD orally; ref. 44). In the subcutaneous setting, tepotinib treatment induced strong tumor shrinkage with complete regressions in 5/5 mice in both models (33, 44). In the orthotopic setting, tepotinib resulted in intracranial tumor shrinkage, as monitored by magnetic resonance imaging, with median tumor volume changes of −84% for LU5349 and −63% for LU5406 (44).

Given that brain metastases are common in *MET*ex14 skipping NSCLC and are associated with a poor prognosis and reduced quality of life (44), there is considerable interest in the use of tepotinib as a CNS-penetrating drug in these patients. Consistent with the preclinical data, intracranial antitumor activity of tepotinib was observed in patients with NSCLC with intracranial metastasis (53–55). Two case reports of patients with *MET*ex14 skipping NSCLC and brain (leptomeningeal) metastases showed marked intracranial tumor responses to tepotinib, with one patient showing disappearance of multiple intracranial metastases within 2 weeks of treatment (across both cases, the cerebrospinal fluid penetration rate of tepotinib ranged from 1.2% to 1.8%; refs. 53, 54). In a third report, in a patient with *MET*ex14 skipping NSCLC and multiple brain lesions, who had received prior crizotinib, chemotherapy, and immunotherapy, pronounced intracranial response to tepotinib was observed, with all brain lesions too small to measure by day 23 (56). Complete response to adjuvant tepotinib has also been reported in a patient with glioblastoma multiforme with *MET*amp (57). Furthermore, in patients with *MET*ex14 skipping NSCLC in the VISION study, tepotinib demonstrated an intracranial disease control rate of 88.4% [95% confidence interval (CI), 74.9–96.1] in patients with target or non-target brain lesions (*n* = 43) and an intracranial objective response rate (ORR) of 66.7% (95% CI, 38.4–88.2) in patients with target brain lesions (*n* = 15), according to Response Assessment in Neuro-Oncology Brain Metastases criteria (58). No significant neurotoxicity has been reported with tepotinib in the clinical setting (55, 59).

## Tepotinib Can Overcome MET-Mediated Resistance to Targeted Cancer Therapies

In metastatic NSCLC, activating *EGFR* mutations are a common oncogenic driver and positive predictive marker for EGFR-TKIs (19). However, *MET*amp is a mechanism of resistance occurring in up to 30% of patients with NSCLC treated with various EGFR-TKIs (1, 2, 19). Under EGFR blockade, *MET*amp provides a bypass resistance mechanism, allowing EGFR-independent activation of ErbB3 and the downstream PI3K/AKT pathway (1). Thus, MET inhibition may help overcome *MET*amp-driven resistance (19).

Preclinical *in vivo* studies demonstrated that tepotinib can overcome EGFR-TKI resistance in NSCLC xenografts harboring *MET*amp (60). In mice bearing patient-derived LU0858 tumors (harboring the *EGFR-*activating L858R mutation and *MET*amp), gefitinib and afatinib had no inhibitory effect on tumor growth whereas tepotinib alone delayed tumor growth significantly, and tepotinib combined with EGFR-TKIs caused complete tumor regression (60). In mice bearing DFCI081 xenografts (harboring *EGFR* Del19 and *MET*amp), tepotinib, alone or combined with EGFR-TKIs (rociletinib, erlotinib, or afatinib), induced complete tumor regression (60). In mice bearing HCC827-GR-T790M xenografts (harboring endogenous *EGFR* Del19, exogenous *EGFR* T790M, and *MET*amp), monotherapy with tepotinib or rociletinib only moderately affected tumor growth, whereas afatinib and erlotinib had no effect. Tepotinib in combination with afatinib or erlotinib also moderately inhibited tumor growth but tepotinib combined with the third-generation EGFR-TKI rociletinib induced complete regression (60). Interestingly, preclinical evidence suggests that some *EGFR*-mutant, *MET*amp NSCLCs may develop exclusive dependence on MET signaling, and so may be amenable to MET inhibitor monotherapy (61). Although clinical data indicate limited benefit of tepotinib monotherapy in this setting (62), characterization of responders may help identify a subset who could be candidates for a monotherapy strategy.

A recent study in preclinical breast cancer models provided evidence that a combination of pan-HER inhibitors with MET inhibitors (including tepotinib) may help overcome HER2 inhibitor resistance among patients with cooperating pan-HER and MET dysregulation (63). A maximal inhibitory effect on HCC1954 breast cancer xenografts was achieved with a combination of HER and MET receptor antagonists (63).

Tepotinib activity has also been evaluated in triple-negative breast cancer (TNBC) cell lines (64). In TNBC, EGFR expression and downstream pathway activation are common; however, anti-EGFR treatments have not been clinically effective (64). As MET is overexpressed in breast cancer (20%–30%), and *MET*amp and MET overexpressions are associated with anti-EGFR resistance in NSCLC, it was hypothesized that MET contributes to anti-EGFR resistance in TNBC (64). In TNBC cell lines, the combination of an EGFR inhibitor (gefitinib or cetuximab) plus tepotinib demonstrated a synergistic anti-proliferative effect, thus targeting that both EGFR and MET simultaneously may provide an effective therapeutic strategy in TNBC (64).

## Rationale for Tepotinib Clinical Dose Selection

Establishing a dose that is pharmacodynamically active and safe is a mainstay in drug development, especially for targeted treatments that are less prone to toxicities. It is therefore essential to develop a thorough understanding of the PK/PD relationship on the basis of *in vivo* preclinical models, to optimize assessment and selection of the clinical dose and schedule. Moreover, the characteristics of *in vivo* models and their predictive value in humans should be established and carefully considered.

For tepotinib, antitumor activity was demonstrated in several studies of xenograft models harboring *MET*amp, *MET*ex14 skipping, and *MET* fusions, as described above (30, 33, 37, 44, 60). Although these models provided preclinical proof of principle for tepotinib, they are limited by their independence from human HGF and their consequent high sensitivity to MET inhibition, which could bias clinical dose selection. To address this, dedicated single- and repeated-dose PK/PD studies were executed in the HGF/MET autocrine KP-4 model, which showed comparably lower tepotinib sensitivity (moderate tumor shrinkage), meaning that higher tepotinib doses were required to achieve maximal response than in very sensitive tumors with oncogenic alterations (43). Thus, the KP-4 model allowed a more conservative estimation of the tepotinib dose–efficacy relationship (43).

Results from these experiments and subsequent PK/PD modeling demonstrated that near-complete (>95%) phospho-MET inhibition for ≥24 hours was required to achieve tumor regression in the KP-4 model (43). After accounting for inter-species differences in plasma protein binding (2.9% in mice and 1.6% in humans), it was estimated that tepotinib concentrations of 390–823 ng/mL in humans were required to attain 90%–95% maximum tumor inhibition. Subsequently, population PK and MET phosphorylation results derived from paired tumor biopsies acquired in the first-in-human (FIH) trial were integrated into the translational model and indicated that ≥95% target inhibition is achieved in >90% of patients with the standard dose of 500 mg (43), and in >80% of patients with a reduced dose of 250 mg (59). On the basis of this model, a biologically active dose of 500 mg QD was defined as the recommended phase II dose (RP2D) for subsequent studies (32).

## Clinical Development Path toward Regulatory Approval in NSCLC

As of December 2022, the tepotinib clinical development program includes 17 phase I and II clinical studies (including clinical pharmacology studies in healthy participants) sponsored by the healthcare business of Merck KGaA, Darmstadt, Germany (see Table 1 for a summary of trials in patients with cancer; refs. 16, 19, 32, 55, 62, 65–71). Of these, 15 studies have been completed or terminated and two phase II studies (VISION and INSIGHT 2) are ongoing. Several investigator-sponsored studies are also underway in patients with cancers with *MET* alterations, including gastric cancer (NCT05439993) and brain tumors (NCT05120960).

**Table 1.**
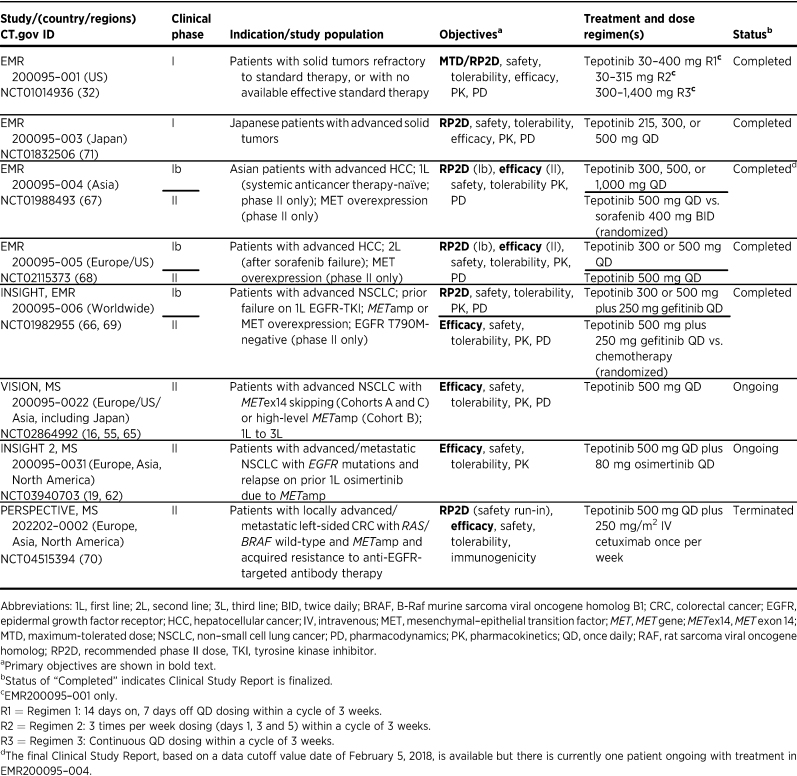
Summary table of completed and ongoing sponsor-initiated phase I and II clinical trials with tepotinib in patients with cancer.

The FIH trial was an open-label, non-randomized, dose-escalation phase I trial in patients with advanced solid tumors conducted to determine the maximum tolerated dose (MTD) and RP2D of tepotinib (NCT01014936; ref. 32). Overall, tepotinib was well tolerated up to the highest dose administered (1,400 mg QD) and demonstrated signs of activity, particularly in tumors with high levels of MET expression or *MET*amp, consistent with the previously described preclinical pharmacology data (32, 72). Although no MTD was identified, an RP2D of 500 mg QD was defined using the translational modeling approach, using preclinical PK/PD data, tumor growth data, and clinical PK/PD data from this trial (32, 43).

Tepotinib underwent further evaluation in patients with tumors with *MET* alterations, including NSCLC with *MET*ex14 skipping or high-level *MET*amp (16, 65), *EGFR*-mutant NSCLC with *MET*-driven resistance to EGFR inhibitors (66), and MET-overexpressing advanced HCC (67, 68). VISION (NCT02864992) is a phase II, multicenter, multi-cohort trial evaluating tepotinib in patients with NSCLC and *MET*ex14 skipping (Cohorts A and C) or high-level *MET*amp (Cohort B; refs. 16, 55, 65). Results from Cohorts A and C demonstrated that tepotinib has robust and durable clinical activity in patients with *MET*ex14 skipping NSCLC (55, 58, 65). In this precision medicine trial, *MET*ex14 skipping was prospectively tested using circulating free DNA (cfDNA) from liquid biopsy (LBx) samples or with an RNA-based approach using fresh or archival tissue biopsy (TBx) samples. Independently assessed ORR was consistent between the LBx (49%; 95% CI, 41–57) and TBx groups (51%; 95% CI, 44–59), underscoring the value of non-invasive LBx testing (73). LBx also enabled collection of longitudinal on-treatment biomarker data, which showed high concordance between the molecular cfDNA response and clinical response based on RECIST (74). In line with another important aspect of the PhAT, baseline and post-progression cfDNA analysis from this trial will be used to understand pre-existing and acquired tepotinib resistance mechanisms, and guide new treatment and combination approaches to reverse resistance (74). VISION data enabled successful regulatory approval of tepotinib for treatment of adults with advanced/metastatic *MET*ex14 skipping NSCLC in several countries. In addition, the trial included a cohort of patients with high-level *MET*amp, *EGFR*/*ALK* wild-type NSCLC without *MET*ex14 skipping, in which tepotinib demonstrated clinically meaningful activity and induced durable responses (16).

The potential for tepotinib to overcome EGFR TKI resistance was evaluated in the phase Ib/II INSIGHT trial (NCT01982955; refs. 66, 69). The randomized phase II part compared tepotinib plus gefitinib versus chemotherapy in patients with *EGFR*-mutant (T790M−negative) NSCLC harboring MET overexpression or *MET*amp, who had failed prior EGFR-TKI therapy. Although no significant difference was seen in the overall population, the tepotinib and gefitinib combination greatly improved outcomes versus chemotherapy in the subgroup with *MET*amp, with a median progression-free survival (mPFS) of 16.6 months (90% CI, 8.3–22.1) versus 4.2 months (90% CI, 1.4–7.0) and median overall survival (mOS) of 37.3 months (90% CI, 21.1–52.1) versus 13.1 months (90% CI, 3.3–22.6), respectively, in the final analysis (69). ORR was 66.7% (90% CI, 39.1–87.7) for tepotinib and gefitinib versus 42.9% for chemotherapy (90% CI, 12.9–77.5). These noteworthy results provided a rationale for the ongoing pivotal phase II study in NSCLC (NCT03940703, INSIGHT 2), evaluating the combination of tepotinib with osimertinib in patients with advanced *EGFR*-mutant *MET*amp NSCLC with acquired resistance to first-line osimertinib (19). Preliminary results indicated promising activity of tepotinib plus osimertinib, with an ORR of 54.5% in patients with *MET*amp by central fluorescence *in situ* hybridization (FISH) testing and ≥9 months’ follow-up (62). Clinical activity of tepotinib plus an EGFR-TKI in NSCLC with EGFR-TKI resistance due to *MET*amp has also been documented in patients receiving this combination outside clinical trials via compassionate use requests (75).

Finally, tepotinib has demonstrated promising activity in patients with HCC with MET overexpression who were either systemic treatment-naïve (NCT01988493; ref. 67) or previously treated with sorafenib (NCT02115373; ref. 68).

The clinical safety of tepotinib and MET inhibitors in patients with *MET*ex14 skipping NSCLC and the recommendations for management of adverse events (AE) have previously been reported in detail (59, 76). Briefly, in patients with *MET*ex14 skipping NSCLC (*N* = 255), tepotinib had a manageable safety profile with a low frequency of treatment discontinuation due to AEs, the most frequent all-cause AEs were mostly mild/moderate and included (overall/grade ≥3): edema [composite term: 69.8%/9.4%, with peripheral edema (60.0%/7.8%) being the most common edema], nausea (26.7%/0.8%), diarrhea (26.3%/0.4%), blood creatinine increase (25.9%/0.4%), hypoalbuminemia (composite term, 23.9%/5.5%), pleural effusions (13.3%/5.1%), vomiting (12.9%/1.2%), and alanine transaminase (ALT) and/or aspartate transaminase (AST) increase (composite term, 12.2%/3.1%; ref. 59). Edema as the most frequent tepotinib AE is a common class effect AE with MET inhibitors and has been reported with capmatinib (all cause peripheral edema: 59.8%), crizotinib (treatment-related edema composite event: 50.7%), and savolitinib (treatment-related peripheral edema: 54.0%; refs. 59, 76, 77). Noteworthy, the reversible increase in blood creatinine levels observed in patients treated with MET TKIs, including tepotinib and capmatinib, is potentially related to competitive inhibition of renal transporters for the secretion of creatinine in the renal tubules (76–78). Tepotinib and its major circulating human metabolite inhibit the elimination of creatinine through inhibition of the organic cation transporter 2 (OCT2) or the multidrug and toxin extrusion (MATE) transporters that could provide an explanation for the observed blood creatinine increase in patients (79, 80). Similar to the inhibition of OCT2 and MATE by tepotinib, other MET TKIs inhibit renal transporters (e.g., capmatinib inhibits MATE1 and MATE2K, crizotinib inhibits OCT1 and OCT2, and savolitinib inhibits MATE1 and MATE2K) and the increase in blood creatinine is potentially a class effect of MET TKIs (76–81). Most AEs can be managed through monitoring, supportive measures, and/or dose reduction/treatment interruption (59, 76).

## Potential Mechanisms of Resistance to Tepotinib

With MET inhibitors now clinically available (26, 27), understanding mechanisms of resistance to tepotinib and other MET inhibitors is of utmost importance to offer alternative treatments to patients who develop resistance. Potential MET TKI resistance mechanisms are heterogeneous (e.g., MET mutations, bypass signaling, mutations in downstream effectors, or histological transformation), and may differ based on the class of MET TKI (22, 82). Besides secondary MET kinase domain mutations (e.g., affecting D1228 or Y1230; Fig. 1F; ref. 31), activation of alternative signaling pathways may result in MET inhibitor resistance, which could be addressed by combination approaches (16, 82). In one *in vitro* study, tepotinib inhibited MET autophosphorylation in 5/8 tested NIH-3T3 cell lines stably expressing mutated MET variants (83). Tepotinib remained active in cells expressing M1268T, H1112Y, H1112L, V1110I, and V1238I mutants, but not in cells expressing Y1248H, L1213V, and V1206L mutants. The tepotinib resistance of the L1213V variant was further confirmed in an *in vivo* xenograft study. It should be noted that this publication adopted an older numbering system for MET amino acids in which the number of each position was increased by 18; hence, Y1248H corresponds to the well-described Y1230H mutation (83). Another study in *MET*ex14 skipping Ba/F3 cells demonstrated that Y1230C/D/S/H/N or D1228A/E/G/H/N/Y kinase domain mutations confer resistance to type 1b MET inhibitors, including tepotinib, capmatinib, and savolitinib (84).

Clinical evidence for these variants as potential resistance mutations was provided by biomarker analyses from VISION. In Cohort A (*MET*ex14 skipping), seven patients had MET codon D1228 or Y1230 mutations at the time of progression (74). More recently, initial biomarker results from Cohort B (high-level *MET*amp) showed emergence of MET kinase domain mutations in 2/9 patients (22.2%) with available end-of-treatment biomarker profiles (D1228H/N/Y, Y1230C/H, and D1231N in one patient, and D1213N, D1228N/H, and Y1230H in the other; ref. 16).

*RAS* mutations may activate the MAPK pathway and could thereby impair the downstream effect of MET inhibition (85). Preclinical data suggest that MET inhibitors combined with MEK inhibitors (i.e., targeting both MET and MAPK) may overcome resistance to MET inhibitors due to *RAS* mutations (85). In EBC-1 cells, tepotinib combined with an inhibitor of Src homology 2 domain-containing phosphatase 2 (SHP2), a cytoplasmic tyrosine phosphatase promoting MAPK pathway activation, delayed emergence of tepotinib resistance, and synergistic inhibition of cell proliferation was observed with tepotinib plus a SHP2 inhibitor in EBC-1, Hs746T, NCI-H1993, and MKN-45 cells *in vitro* (86). Another *in vitro* study suggests that MET inhibitor–induced autophagy may mediate resistance to MET inhibitors, including tepotinib, specifically in gastric cancer models (87). In this study, a combination of MET (tepotinib or PHA665752) and autophagy inhibition (3-MA) in gastric cancer cells significantly decreased cell viability (87).

Thus, a MET inhibitor in combination with another targeted therapy to block alternative signaling pathways may be a strategy for further investigation to overcome MET inhibitor resistance (82, 85–87). Ongoing biomarker studies evaluating pre-treatment and post-progression biopsies will provide further insights into predictive biomarker-directed treatments in patients with MET inhibitor resistance.

## Conclusions

The discovery and development of tepotinib followed a stepwise approach, including the early identification of a robust PD biomarker and development of a deep understanding of the PK/PD relationship with respect to target/pathway modulation, which supported successful clinical dose and schedule selection. Alongside stringent use of biomarker-selected and predictive preclinical models to demonstrate preclinical proof of concept, these studies undergirded a successful clinical development program that enabled approval of tepotinib for advanced/metastatic *MET*ex14 skipping NSCLC. In this respect, the development of tepotinib is fully consistent with the subsequently published PhAT, which describes key questions to be addressed during discovery and development of a molecularly targeted anticancer drug (28). Importantly, the high selectivity and excellent physicochemical profile of tepotinib is a prerequisite for a precision medicine approach.

LBx is a powerful diagnostic tool for precision oncology that has recently been integrated into routine practice and is highlighted as an emerging technology in the PhAT, both to identify appropriate patients for biomarker-driven therapy, and to enable exploratory analysis of resistance mechanisms and alternative response measures (28). Implementation of prospective central testing of *MET*ex14 skipping in LBx samples in VISION significantly accelerated recruitment and generated important data to understand tepotinib resistance (88). Careful analysis of these data will guide rational combination approaches to overcome MET inhibitor resistance in the future.

By selectively targeting MET, the precision medicine tepotinib has shown durable activity in patients with hard-to-treat aggressive NSCLC tumors harboring specific oncogenic *MET* alterations (16, 65). The approval of tepotinib in Japan in 2020 was the first regulatory approval globally for an oral MET inhibitor for treatment of advanced NSCLC harboring *MET*ex14 skipping alterations, and was followed by approvals in multiple other countries/regions (55). Tepotinib is also recommended in clinical guidelines for eligible patients with *MET*ex14 skipping metastatic NSCLC (89–92), and as a treatment option for patients with high-level *MET*amp metastatic NSCLC (91).
